# Exploring developmental factors influencing performance excellence in twice-exceptional Saudi athletes: a case study of Paralympic champions

**DOI:** 10.3389/fpsyg.2025.1556081

**Published:** 2025-04-09

**Authors:** Abdulhamid A. Alarfaj, Marwa M. Hassan, Refah M. Aljohar, Fahad A. Almuaili, Mohamed D. Hassan

**Affiliations:** ^1^Department of Special Education, College of Education, King Faisal University, Al-Ahsa, Saudi Arabia; ^2^Department of Physical Education, College of Education, King Faisal University, Al-Ahsa, Saudi Arabia

**Keywords:** twice-exceptional athletes, Paralympic athletes, elite performance, developmental factors, psychosocial impact, adaptive sports excellence

## Abstract

**Introduction:**

Achieving exceptional performance in sports, particularly among twice-exceptional athletes, is a multifaceted process that remains underexplored. Understanding the developmental pathways that lead to performance excellence is essential for supporting this unique population.

**Methods:**

This study employed a qualitative case study approach involving three male Paralympic champions (mean age: 33 years). Data were collected using achievement portfolios and in-depth interviews to explore the athletes’ lived experiences and identify influential developmental factors.

**Results:**

Two distinct developmental stages emerged from the analysis. The first stage, characterized by spontaneous and unstructured motivation, was shaped by psychological and social influences that sparked a general interest in sports. The second stage involved intentional goal setting and structured practice within specialized sports environments. Key factors such as individual growth characteristics and physiological development influenced each stage differently over time.

**Discussion:**

The findings highlight the complex interplay of personal, psychological, social, and contextual factors in the development of performance excellence in twice-exceptional Paralympic athletes. This research contributes to a deeper understanding of how targeted support and recognition of developmental needs can foster sustained athletic excellence.

## 1 Introduction

Sports play a crucial role in various nations (Khaksar Shahmirzadi et al., 2024; Kuvaldina et al., 2024). Therefore, the development of sports and the focus on the excellence of the athletes have become a top priority (De Bosscher et al., 2009; Fengyingna et al., 2024; Yu et al., 2024). This interest partially stems from the recognition of sports as a significant contribution to the economic growth of lots of countries (Rose and Spiegel, 2011; Khan et al., 2023; Schmid et al., 2023; Teixeira et al., 2023). Moreover, it is among the most popular activities in which large segments of the population participate (Herbold et al., 2020; Jin et al., 2024; Sherry et al., 2024). As a result, many countries aim to boost this impact by developing world-class sporting infrastructure (Sherry et al., 2024).

Countries demonstrate their exceptional abilities in international sports forums and competitions, most notably the Olympic Games, where the world’s top athletes compete (Liao and Pitts, 2013; García et al., 2023; Gómez-Rodríguez et al., 2024). Nations prepare for these events through extensive training programs and qualifying tournaments, aiming to secure the highest number of medals and recognition (Goldblatt, 2016; Humphreys et al., 2018). The global interest in supporting sports for individuals with disabilities has grown significantly. This is largely due to the Paralympic Games, a major international competition where elite athletes with disabilities compete in a variety of specialized sports (Gold and Gold, 2007; Mauerberg-Decastro et al., 2016).

The Kingdom of Saudi Arabia has made notable strides in advancing sports programs under the Ministry of Sports (Alhakami, 2014; Albuhlul, 2022; Sam, 2023; Alghamdi and Aldossari, 2024), particularly in the support and development of athletes with disabilities (Medabesh et al., 2024), including those who are twice-exceptional (Mohammed, 2018). This progress is part of a broader effort to empower and nurture talent by offering specialized training programs aimed at preparing athletes for the Paralympic Games (Reiche and Brannagan, 2022; Loetzner, 2024). These programs were proven to be successful, as Saudi athletes have demonstrated exceptional competitive performances, achieving significant accomplishments at local, regional, and international levels (Sayyd et al., 2020; Albuhlul, 2022). However, despite the success of these programs, additional factors may influence athlete development and achievement, especially considering the psychological, environmental, and societal contexts, as well as the specific circumstances surrounding different types of disabilities.

For example, previous studies have found that athletes experience transitional stages in development and performance improvement based on the experience of graded training (Bruner et al., 2008; Nyhus Hagum et al., 2022; Cury et al., 2024).

Engaging in intensive training during the preparatory phase for Paralympic competitions, combined with effective communication and trust between coaches and athletes, plays a crucial role in understanding the athletes’ unique needs, facilitating the designing of tailored training programs, and meeting the high-performance demands of competitions (Penetrante and Friales, 2023; Dehghansai et al., 2021a,b; Greve et al., 2021; Hafliðadóttir, 2022; Loftus et al., 2022).

Early specialization in sports during childhood and adolescence can hinder long-term growth and development, particularly among elite athletes, due to factors such as physical fatigue, heightened financial burdens, increased injury risk, and limited educational opportunities. In contrast, exposure to a variety of sports during early development, followed by specialization post-adolescence, has been shown to promote long-term sustainability, enhancing both the efficiency and quality of athletic performance over time (Côté et al., 2020; Mosher et al., 2020; Barth et al., 2022; Mclellan et al., 2022; Mosher et al., 2022). Moreover, the number of training hours plays a crucial role in performance improvement, further contributing to the athlete’s overall success (Smith et al., 2017; Barth et al., 2022).

Economic benefits and social context have a significant impact on long-term athletic development (Kaplanidou, 2021; Ribeiro et al., 2021; Rodríguez Macías et al., 2023). Passion and personal inclinations play a prominent role, particularly in studies examining the influence of psychological factors on achievement and performance levels (Dehghansai et al., 2020; Lillegård, 2020; St-Cyr et al., 2021; Alarfaj et al., 2024). Research has shown that passion drives individuals to achieve mastery in performance and active participation, as it is closely linked to emotional factors (Peters et al., 2015; Méndez-Alonso et al., 2021; Askeland, 2022; Alarfaj et al., 2024). Studies have highlighted that emotional factors, such as intrinsic motivation and self-determination, play a pivotal role in encouraging twice-exceptional athletes to pursue and maintain participation in Paralympic sports (Omar-Fauzee et al., 2010; Stanley and Baghurst, 2022; Brown et al., 2023). These factors are also considered key contributors to achieving excellence in athletic performance (Banayan and Georgiadi, 2022; Weinberg and Gould, 2023).

Twice-exceptional (2e) athletes are individuals who possess high athletic potential or competitive excellence, while simultaneously managing cognitive, emotional, or physical disabilities (Alarfaj et al., 2024; Abraham, 2025; Küry and Fischer, 2025), As defined by Alarfaj et al. (2024), twice-exceptional athletes are those with one or more disabilities, diagnosed according to the Saudi Arabian Disability Classification System and World Health Organization (WHO) guidelines. These athletes exhibit outstanding abilities in Paralympic sports and have achieved at least three notable awards at local or international level.

The period spent by elite athletes, from twice-exceptional to being discovered to reaching elite status and excellence in the field, contains different experiences and opportunities that contribute to their growth and development. Therefore, it may be useful to expand the variables when searching for factors and to include more potential variables that might influence them. We searched the educational literature for the factors leading to the development of performance in elite athletes from twice-exceptional to elite. In addition to our reliance on theories based on the results of previous studies, we left the field open for the opportunity to further explore other relevant variables or interacting variables and research as well as research the importance and magnitude of their role in influencing development. In particular, we can benefit from the results of previous and current studies to analyze and interpret them. Developmental changes are tracked and linked with internal and external factors to see how the talents of elite twice-exceptional athletes develop over time and to know which factors are more likely than others to control the rate of this development (Dehghansai et al., 2021a,b; Alarfaj et al., 2024).

In this study, we conducted an in-depth analysis of data obtained from Paralympic athletes, focusing on three exceptional athletes in Paralympic sports. Through this case study approach, we examined their journey from being twice-exceptional (2e) athletes to achieving elite performance and excellence in their respective fields.

## 2 Methods

### 2.1 Study design

The design of this study focuses on gathering data and drawing conclusions to address questions related to individuals who combine disability with exceptional talent in athletics. This was approached through a set of exploratory questions (Cardon and Wilcox, 2011; Kalu and Bwalya, 2017; Sovacool et al., 2018). While this method is not widely employed in gifted education research, the prominence of qualitative studies has grown significantly in recent years (Coleman et al., 2015). Emphasized the importance of synthesizing qualitative research findings related to specific phenomena to gain a more comprehensive understanding of the experiences of gifted individuals and the professionals working to meet their unique needs. In light of these considerations, the researchers selected a multiple case study design (Kahkonen, 2014; Yin, 2018; Alarfaj et al., 2024). The study employed an Explanatory Case Study approach, which aims to explain how events unfold by analyzing cause-and-effect relationships within the data (Yin, 2018; Priya, 2021).

This approach aligns with the study’s objectives and research questions and contributes to the literature in several ways: (a) by tracking the developmental progress of twice-exceptional (2e) individuals and assessing the impact of long-term interventions and programs on their abilities over time, (b) by examining how these individuals respond to changes in both internal and external environments, and (c) by identifying recurring factors across cases and determining their effectiveness in fostering talent development.

Data were collected from individuals at multiple time points retrospectively, and a longitudinal research design was employed to measure changes and key factors that influenced these individuals’ development, ultimately contributing to the emergence of athletic excellence.

### 2.2 Participants

The participants in this study were comprised of three twice-exceptional (2e) sports champions, all male, aged between 24 and 35 years, and citizens of the Kingdom of Saudi Arabia. Written informed consent was obtained from all participants prior to data collection, in accordance with the ethical standards approved by the Deanship of Scientific Research at King Faisal University (KFU-REC-2023-NOV-ETHICS1784).

The study sample was selected using criterion sampling (Kahkonen, 2014; Moser and Korstjens, 2018; Yin, 2018; Priya, 2021). Three primary criteria guided the selection of participants: (1) the individual must have a disability classified under the Disability Law in the Kingdom of Saudi Arabia; (2) the individual must have won medals at the international or Paralympic level; and (3) the individual must still be actively participating in sports at the time of the study (Table 1).

**Table 1:** Participant data.

Sample description variables	First athlete	Second athlete	Third athlete
Name	P1	P2	P3
Age	38	25	36
Condition	Married	Single	Married
Sports type	Discus Throwing, Shot Put	Wheelchair Racing	Running, Cycling
Job	Professional Athlete	Professional Athlete	Private Sector
City	Madinah	Riyadh	Dammam
Athlete classification	Paralympic Athlete	Paralympic Athlete	Special Olympics Athlete
Sport class	F34, T33	T53	Special Olympics Division System (200 m: Div. 43, 400 m: Div. 18)
Sports achievements	Paralympic Silver + World Record (London 2012), Gold (Manchester 2011), Gold in Discus Throw (West Asian Championship 2024), Multiple Asian & Gulf Championship Medals	Paralympic 100 m Gold (Paris 2024), Bronze (Tokyo 2020), Asian Games 100 m Gold (Hangzhou 2022), Multiple World & Asian Medals	Special Olympics Gold (Los Angeles 2015, 200 m), Multiple National & International Medals in Cycling & Running

#### 2.2.1 Disability classification

Participant 1 and Participant 2 are classified as physically impaired athletes, with coordination impairments caused by cerebral palsy. Participant 1 competes in both F34 and T33 classifications, which include seated throwing events such as shot put and discus. Participant 2 competes in the T53 classification for wheelchair racing.

Participant 3 has a diagnosed intellectual disability, confirmed by formal assessments during early schooling, and is classified under the Special Olympics Division SystemP1 is a professional shot put champion, born into a large family of 16 members, including 5 full siblings, and raised in a household of average economic status. At the age of one and a half, he suffered a fall that resulted in triple cerebral palsy, impacting his limbs. Despite these challenges, he pursued education in special schools and completed secondary education with good academic standing. P1’s athletic potential was identified during middle school by his physical education teacher, and his talents were further honed through participation in sports clubs for individuals with disabilities. Over the course of his athletic career, he earned numerous continental medals and secured three Paralympic medals. Notably, P1 holds the world record in discus throw as of the time of this study.

P2 is an accomplished athlete specializing in wheelchair racing for short and medium distances. Born with hemiplegia into a middle-class family, P2 was 5 years old when his father passed away, leaving him to be raised by his mother and four siblings. P2 attended public schools and completed his secondary education before enrolling in university to study business administration. However, he did not finish his university studies, as his athletic career became his primary focus. P2’s athletic talents were discovered through disability sports clubs, leading to numerous successes at local, continental, and international levels. His achievements include gold and silver medals at the World Championships and a medal at the Tokyo Paralympic Games. P2 is also qualified to compete in the upcoming Paris 2024 Paralympics.

P3 is a multidisciplinary professional athlete from the Eastern Province, born into a low-income family with seven brothers, an uneducated mother, and a father holding an intermediate-level qualification. Despite early academic struggles, P3 was eventually diagnosed with an intellectual disability after failing to progress beyond the third grade for six consecutive years. He continued his education in special intellectual education programs, ultimately graduating from high school. P3’s athletic abilities began to shine in primary school, particularly in running, boxing, cycling, and bowling, with the support of his physical education teacher. Over the years, he achieved a considerable number of medals in both short and medium-distance running, as well as in other sports disciplines.

### 2.3 Data collection and instruments

The researchers employed two primary tools for gathering participant data.

#### 2.3.1 Face-to-face in-depth interviews

These interviews began with broad, open-ended questions, gradually narrowing in focus to gather detailed data. The aim was to describe the foundational aspects of the experiences under investigation and identify their core structures, ultimately achieving a comprehensive understanding of the commonalities across participants’ experiences (Creswell and Poth, 2016). The instrument used in the study (currently under publication) consists of 88 items, developed by the researchers, and structured around nine key domains: personal data, social data, economic data, health developmental history, personal and behavioral aspects, academic developmental history, interests and hobbies, and contributions from caregivers. The instrument was reviewed and validated by four university professors specializing in special education, physical education, and psychology.

#### 2.3.2 Analysis portfolio

The initial research instrument, is a model developed for the analysis of sports portfolios, and utilized to identify participants in the study (Alarfaj et al., 2024). This tool is comprised of 54 items organized into five main domains: sporting achievements, nomination and training, psychological characteristics, behavioral traits, and the level of genetic predispositions within the family. The instrument was evaluated and validated by four university professors with expertise in special education, physical education, and psychology.

## 3 Procedures

**Figure 1:**
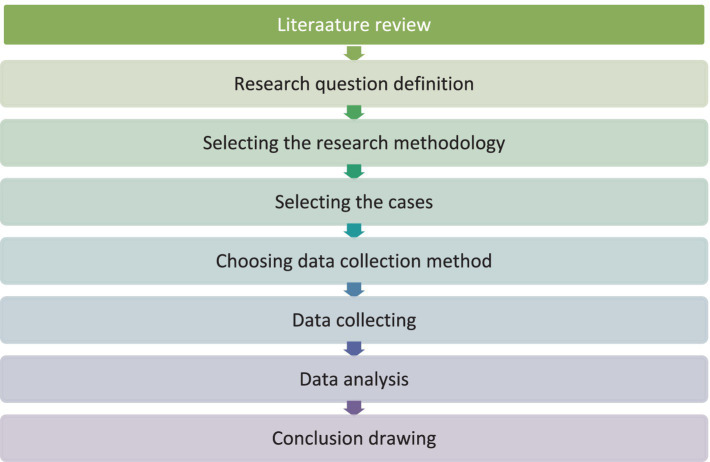
The research process for case studies adapted from Kahkonen (2014).

In developing the case study model, the researchers based their approach on the framework established by Kahkonen (2014). The procedures were delineated into eight sequential stages, as illustrated in Figure 1.

Figure 1 presents the initial stage of the design process, which involves a comprehensive review of the educational literature concerning dual-exceptional individuals within sports contexts. This review aims to assess the existing body of literature and identify pertinent research gaps. Subsequently, the researchers formulated research questions based on these identified gaps and selected an appropriate methodology informed by talent development theory to address these questions. The multiple case study approach, grounded in the interpretive method, was deemed suitable for this investigation.

In the selection of cases, the researchers established specific criteria to ensure the effectiveness of this examination of the lives of Paralympic champions. Following this, the appropriate tools for data collection were identified, with a focus on employing case studies and portfolios as suitable instruments for this purpose. Upon data collection, a qualitative analysis was conducted, utilizing various methods to ensure the credibility of the analysis in accordance with established qualitative scientific frameworks. The data analysis was performed by two members of the research team, whose findings were subsequently reviewed by an independent specialist in the field of physical education. Finally, the results were synthesized and presented in a concise, cognitive map, illustrating the key findings of the study.

### 3.1 Validity

To assess the validity of the case study, the researchers confirmed the structural validity by presenting it to four experts specializing in the fields of special education, gifted education, physical education, and psychology. This evaluation aimed to determine the alignment of the case study with the study’s objectives and the theoretical framework adopted by the researchers (Korstjens and Moser, 2018).

Moreover, the researchers employed multiple data collection tools in addition to the case study model, including in-depth interviews and portfolios (Alarfaj et al., 2024). This triangulation of data sources was designed to enhance the validity of the findings and mitigate the risk of bias. The researchers assigned the interviews from the three cases to different team members to identify triangulating variables effectively. The decision to adopt a multiple case study approach was strategically made to reduce bias and facilitate comparisons between cases, thereby strengthening the basis for data linkage and theoretical development (Kahkonen, 2014).

### 3.2 Reliability

**Figure 2:**
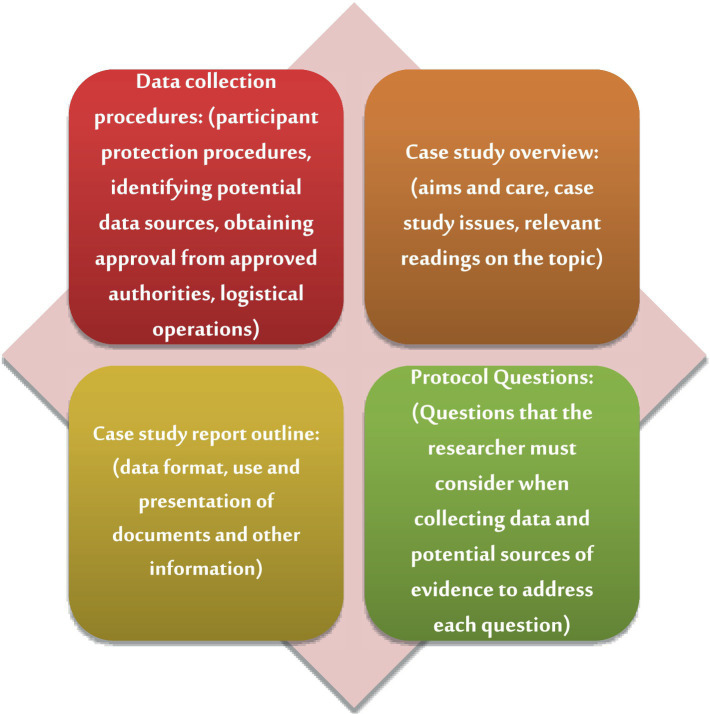
Components of the case study protocol.

To ensure the reliability of the case study, Yin (2018) advocates for the use of a Case Study Protocol. This protocol encompasses an interview guide and establishes procedures for administering the testing tools, alongside a database designed to minimize errors and biases throughout the study. The case study protocol is organized into four distinct sections, as illustrated in Figure 2.

Additionally, the reliability of the qualitative data analysis was ensured by assigning the analysis tasks to two team members independently. Subsequently, the compiled analyses were presented to a researcher who is recognized as a critical colleague, and is specialized in the field of physical education. This collaborative approach aimed to achieve a comprehensive and reliable analysis of the phenomena under investigation, ensuring the findings were both organized and methodologically sound (Korstjens and Moser, 2018; Yin, 2018; Alarfaj et al., 2024).

### 3.3 Data analysis

The researchers conducted interviews with the participants in the case study using the Zoom platform. All interviews were audio-recorded, and detailed notes were taken during the sessions. Following the interviews, the audio recordings were transcribed into written format, with particular attention to nonverbal cues such as laughter, hesitation, and expressions of interest. The three interviews yielded a total of 40 pages of data, comprising of 17,821 words.

Subsequently, two researchers from the team undertook a thematic analysis of the transcribed data. This process involved multiple readings of the data, followed by note-taking, color coding, and the establishment of initial codes based on the primary themes identified in the interviews. The researchers then organized the data into coherent units, identifying patterns, themes, and categories to highlight significant topics related to the lives of champion athletes.

Once the data structuring was completed, the analyses were submitted to an independent expert for further evaluation and refinement, ensuring adherence to the study’s frameworks. This theoretical framework facilitated a comprehensive understanding of the broader context, resulting in the generation of numerous notes, mind maps, and memos. Ultimately, the data was revisited in light of theoretical insights by correlating them with the literature review to substantiate or elucidate the empirical findings.

## 4 Results

In this section, we will present the results derived from our analysis of the experiments. We organized the topics in chronological order to highlight their influence on performance, based on the participants’ recollections. Several key factors contributed to the development of talent and the achievement of professional status in sports. Each factor is detailed below, accompanied by illustrative quotes from the participants (Table 2).

**Table 2:** Key stages for the development of twice-exceptional athletes’ performance.

Stage	Psychological and social factors	Sports factors	Personal and external motivations
1. Early motivation stage	Early maturity and awareness of social requirements	Early detection in line with the sports field	Personal motivation: seeking financial independence and self-fulfillment
	Low economic circumstances of the family	Influence of school activities in sports diversity	Family and community support
	Social and psychological pressures leading to self-reliance	Experiment with various sports.	Self-realization
2. Deliberate practice stage	Acceptance of psychological and health challenges as opportunities	Intensive and targeted training under specialized coaches	Strong desire for sports excellence and achievement
	Psychological resilience through self-control and goal setting	Rigorous, continuous training for accelerated development	Social encouragement and recognition from notable figures
	Consistent family support	Diverse sports experiences leading to the selection of a specific sport	
		Internal and external camps provided by the Olympic and Paralympic Committee	
3. Empowerment and mastery stage	Psychological traits like optimism, patience, and perseverance	Direct guidance from elite coaches for specialized development	Gaining support from leadership and sports clubs
	Ability to handle failure positively	Transitioning to high-level training with top coaches	Support from sports and community leaders for special needs
	Large financial incentives	Continued success boosting confidence and achievement	Awards and recognition from national figures
			Sacrifice in order to achieve athletic excellence

### 4.1 The beginning of motivation to take the sports path

Sports tendencies were not evident in early childhood. However, early disability and personal factors created the ideal conditions for pursuing the athletic path. The participants experienced unintended changes in terms of circumstances and the surrounding environment that were helpful factors in directing them toward the sporting path. The factors that formed the first attitude and motivation can be presented in two groups, the first related to psychological and social situational contexts and the second related to sports situational contexts.

### 4.2 Psychological and social situational contexts

Through the analysis of the participating cases, we realized that there were two contexts that formed the beginning of sports ideas, which are as follows.

### 4.3 Early maturity and awareness of social requirements

The low economic circumstances of the family combined with the conditions of early disability and societal outlook put great pressure on 2e. This may lead them to move beyond childhood and adolescence to maturity and early awareness compared to other children of the same age. We found this clearly in all cases of the study: their discussion of those stages did not tell us about their feelings of reassurance and understanding. In contrast, the stages preceding achievement were full of frustration, psychological problems, and a feeling of inferiority. They had a responsibility to be self-reliant, searching for an alternative that would help them rely on themselves and feel independent and contribute to alleviating the burdens of the family, so the feeling of well-being decreased and the feeling of responsibility increased. P2 mentioned: *We went through difficult circumstances early on. I worked with my brothers in order to get money, and I think this may be one of the reasons that led me to where I am in an early period*. Early maturity served as the fuel that led them to increase their participation in sporting activities. Not only that, but successive achievements as a form of self-realization and fulfillment of social requirements. All participants found an important motivation at the beginning of participating in sports fields, to obtain money. When you can join competitions at an early age, it is possible to participate in competitions and study simultaneously, as this does not pose a great difficulty, unlike working a job, which often require a certain age and other conditions.

Participant P2 stated: When I wanted to join the club and train, my mother refused, but my uncle convinced my mother that this work would bring us a lot of money. Participant P3 stated, I am ready to play in any sport and win tournaments to bring in money.

### 4.4 Psychological and social health

This title may seem contradictory to the above, but despite the difficulties that the participants went through, they, in return, had good psychological health. They accepted everything that happened and considered this an opportunity that could be invested in positive aspects. This helped them control themselves and set their goals accurately. Frequent training and competition entries require many high psychological components to increase the ability to withstand the pressures and challenges that they may face.

Participant P2 described: He does not think of disability as a weakness or a negative point. Rather, he has sufficient psychological capacity to accept this disability and focuses more on athletic excellence.

Participant P1 described the sacrifices he makes to train and attain achievements through adaptation and stated that this adaptation gives him the strength to face all psychological and social difficulties and helps him succeed.

The participants did not talk about the hours they felt regretful but rather expressed them as milestones through which they were able to identify weaknesses and strengthen strengths. P2 said: *Sometimes you may need these challenges to fuel your engine*. Social support concurrent with mental health enhanced continued performance and achievement. Participant P1 described the great role of family support, especially in the beginning, and how appropriate family circumstances shaped his athletic excellence.

### 4.5 Sports situational contexts

We have identified three factors that are considered essential motivators for continuing on the path and achieving mastery of performance. Below is a presentation of these factors.

### 4.6 Diversity of sports experiences in the early stage

The participants did not move directly to a specific sport but rather preceded a phase of experimentation and diversification. P3 stated that he played many sports in early childhood through school activities. Multiple short-term trial periods allowed participants to gain good knowledge about many types of sports and choose the most suitable sport for them. The sport was not chosen for technical and performance factors only; rather, psychological factors were the most controlling factors in determining the appropriate sport.

P2 said: I tried many different types of sports, but I did not find myself in these sports. I am a person who loves enthusiasm and challenge, so I chose the individual sport that gives me this feeling.

### 4.7 Deliberate practice

The deliberate practice that elite athletes undertake with their coaches is one of the factors contributing to the breakthrough developmental changes to reach professional sports. Deliberate practice is not only based on regular training at regular intervals but is also intended to provide trainees with techniques that lead to high levels of performance.

All participants reported that they were subjected to rigorous training by specialized trainers, and these times were not enjoyable for them. However, they expressed their need for this type of training to accelerate their development and win championships. Rather, they considered training to be a type of distinction and reward for them, as only the elite obtain. On the other hand, they found that this was the reason they often won championships, so they found that they had to continue this type of training continuously.

P2 stated: The coach is part of my success. He trains and gives special instructions. He not only performs the tasks needed of him but also helps me in training and achieving my achievements. P1 stated: Intense preparation, practice, and sacrifice. I moved away from my family and moved to another city to train with the trainer, where he is assigned to me. She is a highly experienced and skilled coach. She does not train all athletes but rather is dedicated to the candidates for tournaments. She helped me achieve them.

### 4.8 High level of self-desire to achieve achievement

The feeling of intense desire to achieve achievements began to grow under the influence of two factors: an internal psychological factor, which is the feeling of self-actualization, and an external social factor, which is the appreciation and societal status they find after achieving their achievements. These two factors began to influence in parallel with increased achievement. Participants reported that the inner feeling they feel after achieving success motivates them to achieve another success and overcome all obstacles, and this motivation increases when they receive encouragement and stature in society from prominent figures.

P2 mentioned: When the company held a private party after the World Championship, it was motivating for me, and the minister’s talk with me before the match was inspiring and pushed me to achieve the achievement.

P1 mentioned that he received a valuable gift from King Abdullah before his death, as he gave him King Abdulaziz Medal, which is one of the medals of great status in Saudi society.

This motivation did not face any challenges, as Participant P2 was injured in an accident. The doctor advised against practicing sports, but he insisted on participating and won three silver medals, surprising his doctor.

### 4.9 The stage of mastery of performance and excellence of achievement

After completing the stage of successful beginnings, then came the stage of persistence and mastery of performance. While most of the experiences participants reported were positive, there were no significantly frustrating failures. This success paved the way for the participants to continue their success, raising the level of confidence in their abilities and potential. We have identified the basic factors, according to what participants narrated, that motivated excellence in performance. The following is a presentation of these factors.

### 4.10 Personal and psychological traits and characteristics

All participants showed great awareness of the importance of optimism and good work, and knew how negative behaviors affect their success. They had great openness to life, and good practices reduced psychological negativity and increased positive energy.

P1 said: I participated in more than one tournament and did not achieve anything, and that was very difficult. Everyone came back with medals while I was empty handed, and they were talking about how I had not changed or developed. I did not listen to that and trained myself for 4 years without interruption. I had an intense recovery, and I was patient. Due to financial circumstances, I lived alone in bad housing. After that, I broke a high record in discus throw, and it was a key for me to continue working.

A participant mentioned that he entered a difficult global competition with strong athletes, and some were saying that he would not achieve anything, so he sat far away from people so as to not be affected by negative talk, and, he broke a world record in this game.

### 4.11 Empowerment

The participants expressed that there were several barriers related to empowerment when beginning a career in sports such as their recognition, financial conditions, and training needs, but the stage that came after they achieved some achievements removed these barriers and increased empowerment.

They now have special support from the senior leadership in the clubs, and senior figures in the country give them more confidence, support and endorsement. This allowed them to easily train with the best coaches, provide for special needs, and facilitate their access, all of which supported their athletic progress and increased responsibility on them to achieve success.

Participant P2 stated: The senior leadership in the Ministry of Sports paved everything for him, led by the Minister of Sports.

Participant P1 stated: The support he received from one of the princes played a major role in his success.

## 5 Discussion

Some experimental and qualitative studies have investigated the factors responsible for creating athletic excellence in a specific type of sport (McNamara and McCabe, 2006; Alarfaj et al., 2024), studying the relationships and effects of environmental factors (Storm et al., 2014; Alarfaj et al., 2024), or physiological factors (Aşçi, 2004). However, research into the comprehensive developmental path that leads to extreme athletic excellence has not received a sufficient share of exploration, especially among those with 2e. This prompted us to research this path and benefit from the results of previous studies to explore the methodology leading to talent development and understand the factors influencing the beginning of the path until reaching superior excellence. We tried to explore this through a case study. We communicated with three participants classified as people with disabilities who held world titles and local and international medals in a number of tournaments to understand their experiences and analyze their developmental path to talent.

Each case had a unique sport distinction, but one of the most interesting cases was (P3). This case was classified as having an intellectual disability, although it was one of the most distinctive cases. It may seem on the surface that it is similar to other cases, but the truth is otherwise. He suffered repeated academic failure in the third grade of primary school for 6 years. On the other hand, he had outstanding athletic abilities at an early age that were discovered by the sports teacher. The discovered athletic excellence and the exercises provided by the sports teacher helped rebuild self-confidence and develop positive motivations toward competence and performance (Marks et al., 2013), especially after more academic challenges.

While in the case of (P1), he was classified as having cerebral palsy. However, he achieved records that have not been broken to date, so we cannot say that he is similar to others in the same category. Rather, he has superior athletic excellence that gives him a prominent social status.

In the case of (P2) he also had cerebral palsy and suffered from many challenges, although he won world titles and medals. All cases share living with disability since childhood.

We tried to identify detailed insights into the common factors between these cases that underline the emergence of superior excellence, either in a planned and deliberate manner or due to unintended circumstances. We found that the path to excellence went through two stages: the first stage, the stage of not planning, which represents the fleeting beginning. Then, came the stage of intentional planning, mastery of performance, and excellence of achievement.

The factors of the first stage were obligatory and occurred without prior planning. The individual was also not fully aware of them. However, these factors unfolded in a context conducive to fostering an inclination toward the sports field. Economic difficulties, for example, can create unfavorable conditions by increasing family pressure on children, often forcing them to work to support their families and alleviate financial burdens. This, in turn, reduces their level of well-being and accelerates their maturity compared to their peers (Burton, 2007).

This early maturity led them to search for areas where they could experience joy and a sense of self while making money at the same time. The sports field was the most suitable for them. This is consistent with what was stated in the educational literature, where it was found that poverty in some cases may be an incentive to move toward the sports field because it is cheap and makes the soul happy, and at the same time, it pushes the person to improve performance for the sake of money (Uehara et al., 2021).

The effect of participants’ early access to social awareness of motivation has also been described, and the results indicate a positive effect on performance efficiency (Chan et al., 2023). Thus, we find how positive the consequences were for factors that might seem on the surface to reduce the level of performance. The good psychological state came in conjunction with sports trends. This interaction between mental health and social support contributed to positively resisting challenges. The participants dealt with difficulties logically and considered them as areas that pushed them to make progress.

It has been found that several psychological factors (related to positive personality, motivation, confidence, focus, and perceived social support) protect the world’s elite athletes from the potential negative impact of stressors by influencing their perceived challenge appraisals (Fletcher and Sarkar, 2012).

We have a special understanding of the primary factors leading to the sustainability of work in the same field, which we can call positive adaptation. This adaptation made athletes fall in love with the sports field, and they considered it one of the best outlets for bridging the gaps of excellence due to disability, lack of capabilities and developing the psychological aspects. The multiple experimentation phase was sufficient to confirm the suitability of the chosen field in all psychological and performance aspects. Despite the perceived importance of deliberate and early training in the field and regular practice, there is nevertheless evidence indicating the importance of diversity in practicing different sports, especially in the early stages. This rule is based primarily on psychological, social and emotional aspects, more than its focus on skill and technical aspects. Those who hold this belief justify that experimentation and diversity in practicing sports allows individuals to test themselves, their feelings, their level of motivation, their abilities, and their knowledge in a number of sports (Coté et al., 2009).

Then, they make the appropriate decision based on certainty, Therefore, they were able to adhere to this decision. They focus on the goal. They were also able to bear the burden of the requirements of excellence, which is deliberate practice. The theory of deliberate practice is based on three main assumptions: the first assumption is that it is a factor influencing the development of performance, the second assumption is that it requires great effort, and the third assumption is that it is not enjoyable (Ericsson et al., 1993; Berry et al., 2008).

There is much empirical evidence indicating the effect of deliberate practice on the acquisition and development of skills in individuals. It has been found that its role in reaching the elite is greater than that of domain expertise (Ward et al., 2007), and confirmed by Ericsson et al. (1993).

The role of deliberate practice in reaching the elite stage has two aspects. The first is the occurrence of physiological adaptation, which means the development of many physiological characteristics in the human body as a result of regular and purposeful training over a relatively long period of time. The other aspect is the effect of deliberate practice on memory performance, as it was found that purposeful practice trains memory enough to benefit from the knowledge stored in long-term memory to control exceptional motor performance. Therefore, in a comparison between experienced and inexperienced people, it was found that experienced people excelled in motor synergy compared to others (Alarfaj et al., 2024).

One experimental study also indicated that the continuous development of practice has a positive impact on the feeling of pleasure and provides the necessary motivation to exert appropriate effort as the level of experience increases (Hyllegard and Yamamoto, 2005). This balance between the feeling of pleasure, the development of performance, and the achievement of achievements created a positive adaptation that was able to prolong the psyche of the participants to reach the second stage, in which there was extreme excellence in performance. We found many psychological conflicts, but they supported progress.

Many participants were exposed to events and challenges, but they recovered quickly from them. The reason for this is that their vision has become clear. Belief in one’s abilities has also begun to have a major impact on performance. In addition, they were able to instill confidence in others. These factors came as a system that led to the development of performance. It took place at periods that were proportional to the stages of psychological and physical development in early childhood, and perhaps this is what made its impact positive and gave the participants sufficient energy to advance in performance.

Comparison with Olympic Athletes: The developmental path of twice-exceptional athletes is quite comparable to that of Olympic athletes, despite the fact that they encounter particular difficulties because of their handicap (Alarfaj et al., 2024). Both groups depend on early exposure to a variety of sports prior to specialization in terms of overall sport orientation. Olympic athletes, on the other hand, frequently participate in extremely regimented, systematic training programs from a young age, while 2e athletes might not have access to the same tools and support systems. While healthy athletes usually obtain planned development routes at an earlier age, 2e athletes’ talent discovery is sometimes delayed or ignored because of social assumptions about their skills (Storm et al., 2014).

Disparities are further highlighted by early accomplishments and their assessment. Due to the absence of standardized criteria for gauging their growth, twice-exceptional athletes frequently face more subjective evaluations than healthy athletes, who benefit from established evaluation systems and development programs (Coté et al., 2009). Their long-term success is greatly influenced by the resilience they acquire in overcoming these obstacles.

Lastly, there are notable differences between the two groups’ motivations for quitting sports. While Olympic athletes frequently retire when their career cycles reach their zenith, their performance deteriorates, or they reach old age, twice-exceptional athletes may encounter additional obstacles like limited accessibility, insufficient assistance from sports organizations, or trouble juggling their disability management with high-performance training. Healthy athletes may retire because of injury, declining performance, or personal preference, but twice-exceptional athletes frequently deal with other issues like limited accessibility, a lack of ongoing institutional support, or the physical strain of juggling their disability and competitive demands (Ericsson et al., 1993). Developing more inclusive training programs that address the unique requirements of 2e athletes requires an understanding of these distinctions.

The first stage’s factors happened spontaneously and were mandatory. Additionally, the person was not entirely conscious of them. Nonetheless, these circumstances developed in a way that encouraged a preference for the athletic field. For instance, economic hardship can lead to adverse circumstances by putting youngsters under more familial pressure and frequently requiring them to work in order to support their families and ease financial strains. In contrast to their peers, this consequently lowers their degree of wellbeing and speeds up their maturity (Burton, 2007).

Their early maturity prompted them to look for opportunities to earn money while simultaneously finding happiness and a sense of identity. The best place for them was the sports field. This supports the findings of the educational literature, which found that poverty can sometimes serve as a motivator to pursue a career in sports because it is affordable, fulfills the soul, and encourages an individual to perform better for financial gain (Uehara et al., 2021).

It has also been reported how early access to social awareness of motivation affects participants, and the findings show that it improves performance efficiency (Chan et al., 2023). As a result, we discover how beneficial the outcomes were for elements that initially would appear to lower performance levels. Sports trends coincided with a positive psychological state. This relationship between social support and mental wellness helped people overcome obstacles. The participants approached challenges rationally and viewed them as opportunities to advance.

By affecting their perceived challenge appraisals, a number of psychological factors (associated with positive personality, motivation, confidence, focus, and perceived social support) have been found to shield the world’s top athletes from the possible negative effects of stressors (Fletcher and Sarkar, 2012).

In conclusion, while both Olympic and twice-exceptional athletes share a commitment to rigorous training and competition, the unique challenges faced by 2e athletes necessitate tailored support structures and inclusive policies to ensure equal opportunities for athletic excellence.

## 6 Conclusion

The results of the case study indicated that there are critical stages in the lives of 2e athletes. The stages can be invested well. These stages are divided into two periods, the first in early childhood, which must be the beginning of detection and incitement toward the appropriate sports field and the formation of positive stimuli for primitiveness. An intentional plan should also be built in preparation for the second stage, as we found that the exceptional development of athletes depended largely on the strength of intentional plans, appropriate preparations in various psychological, environmental, and social aspects, appropriate capabilities, and others. In general, when you want to obtain exceptional athletic performance from 2e athletes, you must build a comprehensive development methodology that begins from the early years until they reach beyond-exceptional performance.

## Data Availability

The original contributions presented in the study are included in the article/Supplementary material, further inquiries can be directed to the corresponding author.
