# Supplementary material for: Exploring developmental factors influencing performance excellence in twice-exceptional Saudi athletes: a case study of Paralympic champions
Source: Front Psychol. 2025 Apr 9;16:1556081. doi: 10.3389/fpsyg.2025.1556081 (PMC12014743; doi:10.3389/fpsyg.2025.1556081)
Supplement: Supplementary file 1 [file Data_Sheet_1.pdf]

جامعة الملك فيصل
كلية التربية
قسم التربية الخاصة
برنامج دكتوراه تربية الموهوبين

مسار بطل

Champion's Path

دراسة حالة

Case Study

2024/1445

1. Personal Information

**Name:**		**Code:**		**Age:**	
**Gender:**		**Last Educational Level:**			
**Case Registration Date:**					
**Phone Number:**		**Email:**			
**Region:**		**Governorate:**			
**Referral Source:**					

Summary of Personal Information:

....
....
....

2. Social Information

**Father/Guardian:**	☐ Alive<br>☐ Deceased	**Father's Age:**			
**Father's Education Level:**		**Occupation:**			
**Mother:**	☐ Alive<br>☐ Deceased	**Mother's Age:**			
**Mother's Education Level:**		**Occupation:**			
**Number of Family Members:**		Number of Males:		Number of Females:	
**Birth Order Among Siblings:**					
**Parents:**	Stable	Separated			

- **Siblings' Information**

Summary of Social Information:

....
....
....
....

3. Economic Information

**Do you have a fixed income?**	Yes	No		
**Source of income:**	Employee	Ministry of Social Development	Family	Other
**If the source of income is employment:**	Government Employee:	Private Sector Employee:		

Father's income level:	1,000-4,000	5,000-9,000		More than 9,500
Mother's income level:	1,000-4,000	5,000-9,000		More than 9,500
Type of housing:	Apartment	Traditional House	Villa	Other
Availability of transportation:	Personal Car	Public Transportation	Relatives	Other
Housing ownership:	Owned	Rented	**Gifted**	

Summary of Economic Information:

....

....

....

4. Health Development History

Disability:	Autism Spectrum Disorder	Physical Disability			
Diseases and injuries experienced during developmental stages:					
Current general health status:					
Early motor development : Crawling		Sitting		Walking	
Sleeping habits: (Normal)		Unstable			
Nutrition:					
Completion of basic vaccinations:					
Genetic diseases in the family (if any):					
Developmental problems experienced by the student:					
Hearing test:	Normal	Hearing Impaired	Deaf		
Vision test:	Normal	Visually Impaired	Blind		
Neuromuscular balance:	Normal	Weak	Unstable		
Hyperactivity and impulsivity	Normal	Low	High		
Medical history					

Disease or injury	Age	Duration	Extent of Impact	Source of Information

Summary of Health Development History:

....

....

....

5. Personal and Behavioral Aspects	Always	Often	Sometimes	Rarely
Self-Confidence				
Self-Esteem				
Attention to Appearance				
Sense of Responsibility				
Leadership Tendency				
Feeling of Anxiety				
Emotional Stability				
Introversion				
Aggressiveness				
Friendship Formation				

Summary of Personal and Social Development:
....
....
....
....

6. Academic Development History				
Highest Academic Qualification Obtained:	Intermediate or below	Secondary	University	Postgraduate Studies
Do you have an academic degree in the sports field?	Yes		No	
Have you attended courses or workshops in the sports field, and how many hours?	Yes		No	
Sources of training in the sports field:	Association	Educational Institution	Other	
What are the sources of self-learning (if any)?	Books	Works	Digital Resources (Internet)	
Have you experienced learning difficulties?	Yes		No	
Subjects of difficulty (if any):				
Have you received excellence certificates?	Yes		No	
Number of academic excellence certificates (if any):				
Number of school sports participations (if any):				
Attitude toward studies:				
Have you joined school activity groups?	Yes		No	

Activity group during the three stages:	Primary School: ………………	Middle School: ……………………	Secondary School: ……………………	:University …………………

Stage	Placement Location	Subjects of Excellence	Subjects of Weakness	Number of Repetitions
Primary School				
Middle School				
High School				
University				

Summary of Academic Development History:

………

………

………

7. Interests and Hobbies

Academic Interests:	
Sports Interests:	
Artistic Interests:	
Ability to Use the Internet:	
Preferred Social Media Platforms:	
Other Hobbies:	
Influential Factor in This Inclination:	

8. Discovery and Excellence

Talent Discoverer (Individuals who contributed to identifying the talent):	
Mentor or Supporter for Talent Development (Individuals who provided support and care for the talent):	
To what extent did the family contribute to discovering your talent?	
Fields of Sports Excellence:	
Have you undergone any sports tests, and what are they?	
First Achievement Accomplished:	
Competitions or Championships Participated In:	
Do you possess excellence in other areas, such as (calligraphy, sculpture, photography, digital arts, etc.)?	
Obstacles that delayed the discovery of your talent:	
What are your future aspirations in your field?	
Talent Discoverer (Individuals who contributed to identifying the talent):	

Mentor or Supporter for Talent Development (Individuals who provided support and care for the talent):	

Summary of Case Discovery:

………
………
………
…………………………………………

9. Level of Genetic Indications in the Family

Number of Family Members Involved in the Same Sports Field or Other Sports Fields (if any):	
Degree of Kinship (if there are athletes in the family):	
Age at Which the Talent Was Discovered:	
Time of Receiving Training and Development:	
Duration Needed to Master Sports Skills:	
Number of Achievements Accomplished:	
Level and Scope of Achievements:	
Level of Sports Ability (Amateur - Professional):	
Age at Which Relatives Stopped Practicing Sports (if applicable):	

Summary of Genetic Indications in the Family:

………
………
………
…………………………………………

10. Institutional Support:

Role of Educational Institutions:	
Role of the Family:	
Role of Community Institutions:	
Role of the Private Sector:	
Role of the Ministry of Sports:	
Role of Sports Clubs:	
Sources of Financial Support:	
Provision of Coaches:	
Provision of Sports Equipment:	
Availability of Training Facilities:	

**Provision of Suitable Transportation:**	
**Availability of Necessary Medical Care (if needed):**	

Summary of Talent Support:

....
....
....
....

**Decision on Case Participation in the Study:**	

Case Study Team:

Case Study Conductor:	Data Analyst:	Reviewer:
Name:	Name:	Name:
Signature:	Signature:	Signature:
